# The Immunoglobulin Superfamily Members *syg-2* and *syg-1* Regulate Neurite Development in *C. elegans*

**DOI:** 10.3390/jdb10010003

**Published:** 2022-01-09

**Authors:** Dana K. Tucker, Chloe S. Adams, Gauri Prasad, Brian D. Ackley

**Affiliations:** 1 The School of Natural Sciences, University of Central Missouri, Warrensburg, MO 64093, USA; dtucker@ucmo.edu (D.K.T.); gcp39750@ucmo.edu (G.P.); 2 Department of Molecular Biosciences, University of Kansas, 5004 Haworth Hall, 1200 Sunnyside Ave, Lawrence, KS 66045, USA; cadams27@uw.edu

**Keywords:** SYG-2, Ig superfamily, Wnt signaling, axon guidance, axon initiation

## Abstract

Neurons form elaborate networks by guiding axons and dendrites to appropriate destinations. Neurites require information about the relative body axes during the initial projection from the cell body, and failure to receive or interpret those cues correctly can result in outgrowth errors. We identified a mutation in the Ig superfamily member *syg-2* in a screen for animals with anterior/posterior (A/P) axon guidance defects. We found that *syg-2* and its cognate Ig family member *syg-1* appear to function in a linear genetic pathway to control the outgrowth of GABAergic axons. We determined that this pathway works in parallel to Wnt signaling. Specifically, mutations in *syg-2* or *syg-1* selectively affected the embryonically derived Dorsal D-type (DD) GABAergic neurons. We found no evidence that these mutations affected the Ventral D-type neurons (VD) that form later, during the first larval stage. In addition, mutations in *syg-1* or *syg-2* could result in the DD neurons forming multiple processes, becoming bipolar, rather than the expected pseudounipolar morphology. Given SYG-2′s essential function in synaptogenesis of the hermaphrodite-specific neurons (HSNs), we also examined DD neuron synapses in *syg-2* mutants. We found *syg-2* mutants had a decreased number of synapses formed, but synaptic morphology was largely normal. These results provide further evidence that the GABAergic motorneurons use multiple guidance pathways during development.

## 1. Introduction

Nervous system development is a complex process that is, to a degree, orchestrated by secreted and cell surface-associated molecules that work together to achieve the finished product. During the early stages of development, neurons form processes, often called neurites, which can develop into either axons or dendrites. Neurites must extend away from the cell body and be guided into appropriate target regions to form synapses and, ultimately, functional neural networks. Understanding how discrete guidance molecules work together to instruct the elaborate complexity observed in mature nervous systems is a key goal of developmental neurobiology.

*Caenorhabditis elegans* have a highly stereotyped nervous system, providing a robust background to identify guidance molecules, even if the phenotypic penetrance is quite low. For example, we previously showed that mutations in *fmi-1,* the single *C. elegans* Flamingo/Celsr ortholog, can result in a low-penetrance phenotype called a posteriorly directed neurite (Pdn), where axons that should extend anteriorly are instead projected posteriorly [1]. Specifically, in *fmi-1* mutants, Pdns form exclusively in the Ventral D-type (VD), but not the Dorsal D-type (DD), GABAergic motorneurons [1]. In this context, FMI-1 functions cell non-autonomously to regulate GABAergic neuron development [1]. 

Flamingo is a member of the cadherin family of cell adhesion proteins, unique by having a seven-transmembrane domain [2,3]. Flamingo-like cadherins are evolutionarily conserved and have been found to function in epithelial and neuronal development in multiple systems. For example, axon guidance along the anterior/posterior (A/P) axis is regulated by Celsr3, one of the vertebrate *fmi-1* orthologs [4]. In addition, Flamingo-like proteins have been shown to function in parts of the planar cell polarity (PCP) pathway, by regulating the asymmetric localization of PCP components inside cells, including Frizzled, a key component of the Wnt signaling pathway [5,6,7,8]. Finally, Flamingo-like proteins are involved both in early neural development, e.g., axon outgrowth, and later events, including synaptogenesis and synaptic maintenance [9,10,11]. 

Previously, we discovered that Pdn defects in *fmi-1* mutants are synergistically enhanced by mutations in Wnt signaling pathway genes, suggesting that they function in parallel genetic pathways. Genetic interaction between *flamingo* orthologs and components of the Wnt signaling pathway in neuronal development appears to be conserved evolutionarily [12,13,14,15]. In some contexts, Wnts and Flamingo are compensatory, and in others, they are antagonistic. For example, in the early embryogenesis of zebrafish, Celsr1 works in concert with Wnt11 [16], whereas in synaptic development, Wnt5a inhibits synaptogenesis dependent on Celsr3 [14]. Thus, we need to apply contextual information in each case to elucidate how Wnts and Flamingo proteins function.

To better understand the molecular events that participate in anterior/posterior axon outgrowth, we conducted a forward enhancer genetic screen in *mig-5*/Disheveled mutants for animals with Pdns. One of the alleles we recovered was a nonsense mutation in *syg-2*, which was previously identified in a screen for synaptogenesis defects in *C. elegans* [17]. *syg-2* is an evolutionarily conserved member of the Ig superfamily (IgSF) of cell adhesion molecules. This group of molecules is characterized by the presence of immunoglobulin (Ig)-like motifs, often in the extracellular domain, where they exhibit homophilic or heterophilic interactions, typically, but not exclusively, with other IgSF proteins. In *C. elegans*, SYG-2 acts as an epithelial guidepost protein, mediating synaptic location specificity by binding to the IgSF member SYG-1, which functions in the hermaphrodite-specific neurons (HSNs) [18,19]. Mutations in *syg-1* also synergized with loss of *mig-5* in A/P outgrowth, consistent with *syg-1* and *syg-2* functioning together in this context. This work describes a new role for the Syg genes, specifically regulating axon outgrowth in parallel to Wnt signaling to prevent the formation of ectopic secondary neurites in DD neurons. In contrast to previous reports of *syg-1* and *syg-2* dramatically affecting HSN synapse formation, we found a mild effect of *syg-2* loss-of-function mutations on synaptogenesis in GABAergic neurons. Our results suggest that these proteins likely function differently in these neuron types.

## 2. Materials and Methods

### 2.1. Strains and Genetics

N2 (var. Bristol) was used as the wild-type reference strain in all experiments, and CB4856 was used for polymorphic mapping of *lh6*. Strains were maintained at 18–22 °C, using standard maintenance techniques as described [20]. Alleles used in this report include: *LGI—lin-17(n671); LGII—mig-5(rh94); mig-5(tm2639); dsh-1(ok1445); LGV—fmi-1(tm306); LGX—syg-2(ky673); syg-2(lh6); syg-1(ky652)*. The following integrated strains were used: *LGII—juIs76* [*Punc-25::gfp*]; *juIs1* [*Punc-25::snb-1::gfp*]; and *lhIs47* [*Punc-25::mCherry*]. 

### 2.2. EMS Mutagenesis and Genome Resequencing

Approximately 20–25 P0 *mig-5(rh94)juIs76* L4 animals were mutagenized using 50 mM ethyl methanesulfanate (EMS) in M9 for four hours as described [20]. Approximately 2500 F1 mutagenized animals were individually plated and allowed to self-fertilize, and F2 offspring were scored for an incidence of Pdns > 2% using a Leica MZ9.5 fluorescence stereo dissecting microscope. After isolation, animals were outcrossed to an unmutagenized *mig-5juIs76* parent and then re-isolated prior to CB4856 mapping.

CB4856 males were crossed to the *mig-5juIs76*; *lh6* mutants, and then ~20 F2 animals with Pdns were single plated and allowed to have progeny. Genomic DNA was isolated from the populations and then mixed and prepared for sequencing by the KU Genome Sequencing Core. FASTQ files were processed on the Galaxy server (usegalaxy.org) using the CloudMap pipeline [21,22]. The *lh6* mutation creates a stop codon that was verified by PCR and Sanger sequencing.

### 2.3. Imaging and Analysis

Imaging was conducted using an Olympus FV1000 laser scanning confocal microscope with the Fluoview software. Images were exported to ImageJ for analysis as described [1]. Synaptic puncta were imaged and quantified as described, with the following modification: the threshold was set automatically using the Otsu method [11,23]. To differentiate whether double mutants exhibited a synergistic or additive phenotype, we used the following formula to estimate an additive model [Phenotype 1(%) + Phenotype 2(%) − (Phenotype 1(%) × Phenotype 2(%)]. Fisher’s exact test was calculated with Prism GraphPad (5.0) to determine whether observed values were significantly different from an additive model. In those calculations, we set a threshold of *p* < 0.005 to determine significance, to account for multiple testing. ANOVAs were performed in R [24] to determine if genotype, allele or animal stage contributed to the variance observed, comparing between double and triple mutants using R, with a Tukey honestly significant difference test to conduct pairwise comparisons. All genotypes were scored on a minimum of two different days, and the results were averaged between scoring sessions. 

### 2.4. Data Availability

All strains presented are available upon request.

## 3. Results

### 3.1. syg-2 Mutations Cause Pdns

**Figure 1:**
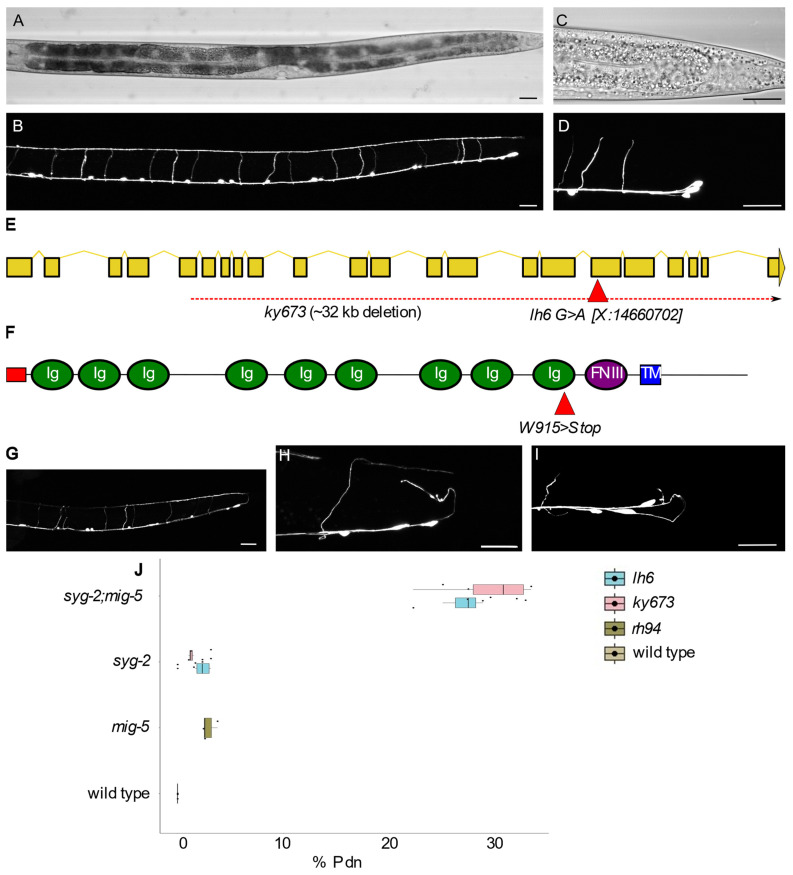
*syg-2* mutants exhibit posteriorly directed neurites (Pdns). (**A**,**B**) A wild-type *juIs76* animal ((**A**)—transmitted light) and GFP ((**B**)—GFP). For all images, anterior is left and dorsal is upward, and scale bars = 20 μm. The DD and VD neurons form a ladder-like presentation, with regularly spaced commissures forming the rungs (**B**). (**C**,**D**) In the posterior region of the animal, four D-type neurons are visible. At the end of the ventral nerve cord are the VD12, DD6 and VD13 neurons in a cluster. Note that no processes extend to the posterior of this cluster. (**E**,**F**) A schematic of the *syg-2* gene (**E**) and protein (**F**), with the location of the *lh6* mutation indicated. (**G**–**I**) Pdns in *syg-2(lh6), syg-2(ky673)* and *syg-2(lh6)*; *mig-5(rh94)* mutants are presented, respectively. (**J**) Boxplot of the percentage of animals observed with Pdns by genotype.

In *C. elegans*, the GABAergic primary motorneurons provide inhibitory input to the body wall muscles during locomotion. Two distinct classes of GABAergic motorneurons form at different times of development and arise from distinct cell lineages [25]. The Dorsal D-type (DD) GABAergic motorneurons are born early in embryogenesis, while the Ventral D-type (VD) GABAergic motorneurons form during the first larval stage. In spite of their unique lineage and period of generation, the neurons are morphologically similar. There are 19 GABAergic motorneurons, and each is pseudounipolar. That is, they form a single process that bifurcates into a region specialized for presynaptic components (axonal) and a separate region that forms postsynaptic structures (dendritic). The D-type neurons generally share a characteristic “H-shaped” morphology with a cell body along the ventral midline of the animal, and a single process that extends anteriorly, forming a commissure and processes that extend along both the ventral and dorsal nerve cords (Figure 1B) [26]. 

The highly stereotyped pattern of GABAergic motorneurons makes it straightforward to identify mutations that cause developmental defects. Previously, we described a phenotype whereby the axons emanating from the cell body inappropriately projected toward the posterior of the animal, instead of the proper anterior direction, and termed this phenotype posteriorly directed neurite (Pdn) [1]. Although the incidence of Pdns was roughly equivalent across the neurons, it is easiest to score Pdns in the most posterior region of the animal. The last three GABAergic neurons (VD12, DD6 and VD13) form a cluster (Figure 1D), and the observation of any axons found posterior of those cell bodies can be classified as a Pdn axon outgrowth error.

To better understand the molecular cues that guide anterior/posterior neurite formation, we performed an EMS mutagenesis screen in *mig-5*/Disheveled mutants, which have a low level of Pdns (~2%) but can be enhanced by mutations in other genes, including *fmi-1* [1]. We isolated an allele, *lh6,* where the incidence of Pdns in the progeny was ~25%. We used polymorphic mapping (to CB4856), genome resequencing and the CloudMap pipeline to determine that *lh6* created a nonsense mutation in *syg-2*. SYG-2 is a member of IgSF cell adhesion molecules. The protein is generally organized into an extracellular region comprised of Ig-like domains, a single-pass transmembrane domain and an intracellular region. Although the intracellular domain seems to lack any predicted motifs, it is required for intracellular trafficking [19]. 

RNA sequencing predicted seven different SYG-2 isoforms, ranging in size from 1230 AA (isoform B) to 95 AA (isoform C). Aside from the C isoform, which would lack the entire extracellular region and transmembrane domain, the predicted isoforms differ in the number of Ig domains present but contain the same transmembrane and intracellular domain. The *lh6* mutation affects nucleotide X:14660702 (WormBase version 280), causing a W915Stop mutation (SYG-2B isoform) (Figure 1E). Based on the location, this stop codon is expected to impact all *syg-2* isoforms, except isoform C.

We confirmed that *lh6* was an allele in *syg-2* when it failed to complement another *syg-2* mutation, *ky673* [17]. The *ky673* allele is a deletion that starts within the *syg-2* coding region and removes portions of four genes along the X chromosome, including the entire predicted *syg-2C* isoform, and thus is likely a molecular null for the gene. We then determined the rate of Pdns in *syg-2* (Figure 1G,H) and *syg-2; mig-5* (Figure 1I), using both the *lh6* and *ky673* alleles (Figure 1J, Table 1). We observed Pdns in ~1.5% of the *syg-2* single mutants, as both the *lh6* and the *ky673* alleles acted equivalently. In the *syg-2; mig-5* double mutants, the rate of Pdns (27%) was significantly higher than the single mutants alone (Table 1) or a predicted additive model (~3.5%). Using an ANOVA, we found a significant difference in the genotypes, but no significant difference based on the *syg-2* allele used, nor an interaction between genotype and allele (Supplemental Table S1). Based on these results, we conclude that *lh6* creates a complete loss of function in *syg-2,* that mutations in *syg-2* result in a low level of Pdns and that *mig-5* loss-of-function mutations synergistically enhanced Pdn formation in *syg-2* mutants.

We examined the animals for other types of developmental defects. At a gross level, we found no obvious differences in the number of GABAergic motorneurons formed (19 cells/animal). Since our transgene is a GABA-specific marker (*Punc-25*::GFP) [27,28], these observations indicated no obvious errors in cell division or differentiation were occurring. Further, the positioning of the neurons was grossly normal, suggesting cell migration errors were not common, although in some animals, the VD12, DD6 and VD13 neurons were not clustered. We have previously documented that the formation of Pdns is independent of whether those neurons cluster [1]. Finally, we noticed some additional guidance defects, e.g., left/right outgrowth errors, as we and others have previously described [11,28,29]. However, such guidance errors would have been subsequent to the initial outgrowth from the soma and were not investigated further in this report. 

### 3.2. syg-2 and syg-1 Function in a Common Pathway in Parallel to Wnt Signaling

**Figure 2:**
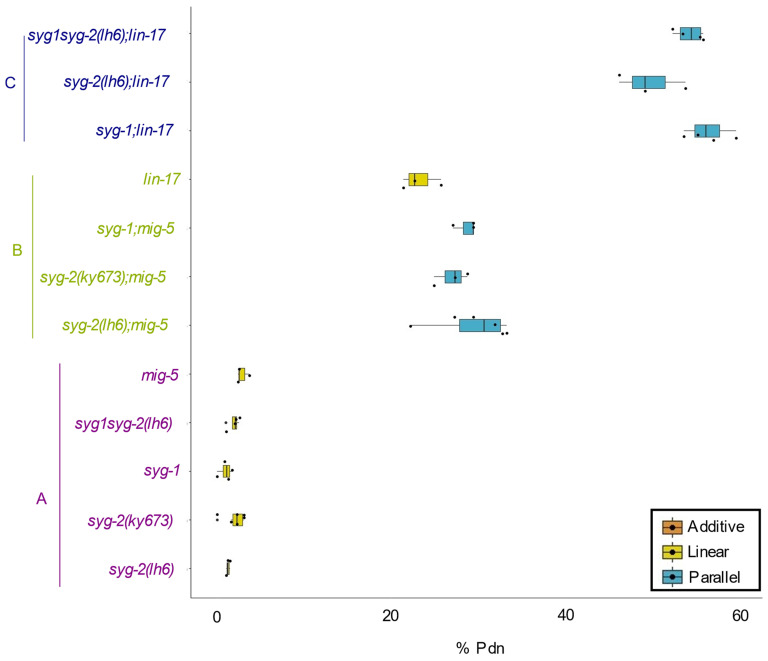
The Syg and Wnt pathways function in parallel. The percentage of animals displaying a Pdn is indicated by the boxplot. There are three significance groups (**A**–**C**). Each genotype within a group is not significantly different from the others in the same group but is significantly different from all genotypes in the other groups by pairwise comparisons. The color of the boxplot demonstrates the type of genetic interaction concluded by genetic analysis.

In most contexts, SYG-2 functions with the IgSF protein SYG-1 [18]. We asked if this was true in the context of Pdn formation in D-type neurons. We obtained an allele of *syg-1, ky652,* and scored Pdns in those animals. We observed that the *ky652* animals exhibited Pdns at a rate of 1.5%. We found that *syg-1; mig-5* animals exhibited Pdns at approximately the same frequency as *syg-2; mig-5* (Figure 2, Table 1). Further, significance testing indicated that the *syg-1syg-2* animals were indistinguishable from the *syg-1* or *syg-2* single mutants (Supplemental Table S2).

We also examined the interaction between *syg-2*, *syg-1* and *lin-17* which encodes one of the four Frizzled-like Wnt receptors [30]. We found that *lin-17* single mutants had a higher rate of Pdns than *mig-5* alone (Figure 2, Table 1). However, we found synergistic increases in Pdn formation in both the *syg-2;lin-17* and *syg-1;lin-17* double mutants, with approximately the same rate of increase (~27%) from *lin-17* alone. This difference was similar to the synergy found in the Syg gene double mutants with *mig-5.* Finally, we were able to make *syg-1syg-2;lin-17* triple mutants, which were indistinguishable from the *syg-2;lin-17* or *syg-1;lin-17* double mutants (Supplemental Table S2). Taken together, all of these results are consistent with *syg-1* and *syg-2* functioning in a single genetic pathway that functions in parallel to Wnt signaling to regulate GABAergic neurite development in *C. elegans*. 

### 3.3. syg-2 and syg-1 Mutations are Additive with fmi-1

**Figure 3:**
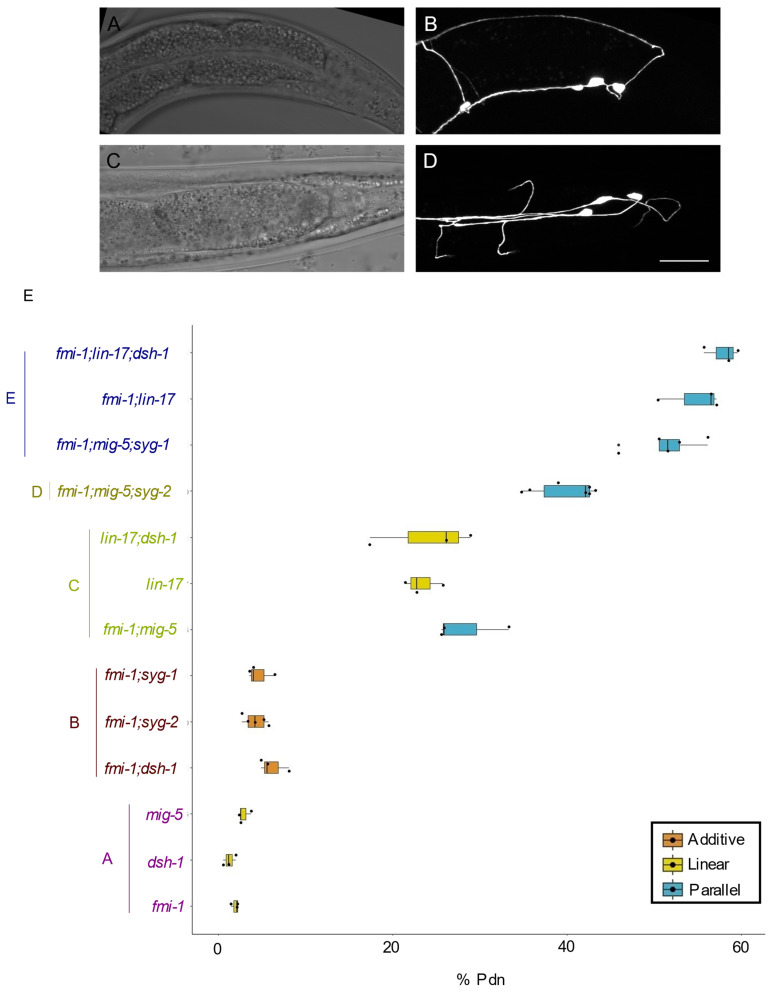
Genetic interactions between the Syg, Fmi and Wnt pathways in Pdn development. (**A**–**D**) Representative images of the percentage of *fmi-1;mig-5;syg-2* animals displaying Pdns. (**A**,**B**) Lateral view. (**C**,**D**) Ventral view. (**E**) Boxplots of Pdns observed by genotype. Significance groups are color coded and indicated by letters. Each genotype within a group is not significantly different from the others in the same group but is significantly different from all genotypes in the other groups by pairwise comparisons. The color of the boxplot demonstrates the type of genetic interaction concluded by our analysis.

Previously, we demonstrated that *mig-5* or *lin-17* loss of function enhances the rate of Pdn formation in animals lacking the *C. elegans* Celsr/flamingo ortholog *fmi-1* [1]. The incidence of Pdns in *mig-5; fmi-1* double mutants was similar to that we observed in the *syg-2; mig-5* double mutants (Table 1). Thus, we hypothesized that *syg-2* might function in the *fmi-1* genetic pathway. We generated *syg-2; fmi-1* double mutants (Figure 3B,D), and we determined that the rate of Pdn formation was consistent with an additive model, rather than synergistic (Figure 3E, Table 1), which suggested these genes functioned in independent pathways. We confirmed this by creating *syg-1; fmi-1* double mutants, which again had an apparent additive interaction (Figure 3E, Table 1). We also created *fmi-1; mig-5; syg-2* triple mutants. In those animals, we observed an additive rate of Pdn formation, compared to either the *fmi-1; mig-5* or *syg-2; mig-5* double-mutant backgrounds. The same was true for *fmi-1;mig-5;syg-1* triple mutants, which exhibited a synergistic enhancement, which was even higher than that of *fmi-1;mig-5;syg-2* (Figure 3E, Table 1). Taken together, these results suggest that while *mig-5* is an enhancer of both the *syg-2* and *fmi-1* backgrounds, there was no obvious cross-talk between the Syg and Fmi pathways.

The penetrance of Pdns in *lin-17/Frizzled* mutants was generally higher than that of *mig-5/Disheveled.* Thus, we asked whether a second *Disheveled* gene, *dsh-1,* was functionally compensating when *mig-5* was mutated. Using a deletion that removes part of the *dsh-1* coding region (*ok1445*), we observed a low incidence of Pdns (~1.3% of animals). We observed Pdns in 35% of the adults in *mig-5dsh-1* double mutants (Table 1). *lin-17;dsh-1* exhibited Pdns 24.2% of the time (Table 1), which was indistinguishable from *lin-17* alone (Supplemental Table S2). These results indicate that *mig-5* and *dsh-1* function in a compensatory manner, but that *dsh-1* likely functions in a linear pathway with *lin-17.*

To determine if *dsh-1* functioned in the *fmi-1* pathway, we created double mutants with *fmi-1.* Those animals had a slightly higher incidence of observed Pdns (6.2%) than might be expected in an additive pathway (3.2% calculated), but below what we had generally seen in synergistic interactions. We confirmed this by examining *fmi-1;lin-17* double and *fmi-1; dsh-1; lin-17* triple mutants (Table 1). In each case, our data are consistent with *dsh-1* being in the same pathway as *lin-17,* and in parallel to *fmi-1,* as previously described [1].

### 3.4. syg-1 and syg-2 Primarily Affect DD Neurons, While fmi-1 Affects VD Neurons

**Figure 4:**
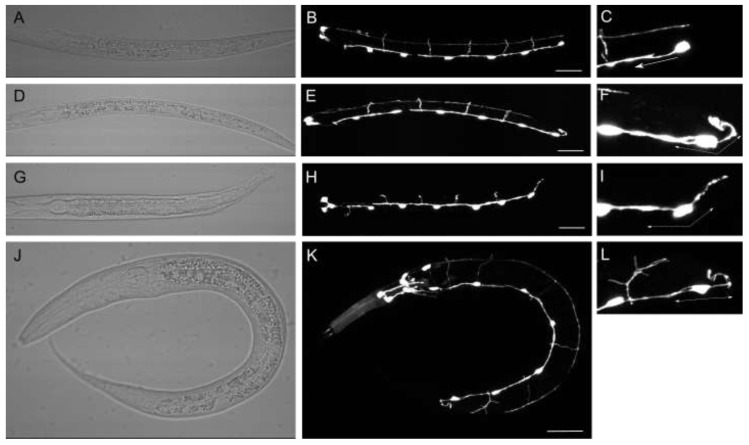
*mig-5* and *syg-2* L1s have bipolar Pdns. (**A**–**C**) Wild-type animals have 6 DD neurons (arrowheads). (**C**) DD6 extends its axon in the anterior direction (arrow). (**D**–**F**) *syg-2(lh6)* animals with Pdns have DD6 neurons with two processes. F) DD6 extends one axon anteriorly and one dorsally (arrows). (**G**–**I**) A *mig-5(rh94)* L1 exhibits a bipolar DD6. (**J**–**L**) A *mig-5; syg-2* double-mutant L1. The DD6 processes extend anteriorly and posteriorly. Note that the anterior commissure forms multiple branches. Scale bars are 20 μm.

There are two classes of GABAergic primary motorneurons, DDs and VDS. Since the DD neurons form during the first larval stage (L1), prior to the formation of the VD neurons, we can specifically score the DD neurons with single neuron resolution in L1 animals (Figure 4A–C). We found that, in *syg-2* or *syg-1* mutants, the penetrance of Pdns was equivalent in L1s or adults (Figure 4D–L, Table 2), suggesting that the DD neurons were forming Pdns in these mutant animals. Consistent with our previous results, there were no Pdns in *fmi-1* L1 mutants [1], confirming that the Pdns are from VD neurons. We further found that L1 animals of double mutants between *sgy-2* or *syg-1* and *fmi-1* were indistinguishable from L1 *syg* single mutants alone. 

Interestingly, we found a slightly higher rate of Pdns in *mig-5* L1 animals compared to the incidence in adults (Table 2). Whether those Pdns are transient or some of the animals with Pdns die as larvae is unclear, but this was the only mutant background where such a result was observed (i.e., higher incidence in L1s than adults). Conversely, the rate of Pdns in the L1s of *lin-17* mutants (2.7%) was significantly lower than that observed in adults (23.3%). We found no Pdns in the L1s of *dsh-1* mutants (Table 2). The L1s of *dsh-1mig-5* double mutants had a higher than expected incidence of Pdns (27%), when compared to the single Disheveled mutants. We also observed synergistic increases in all of the combinations of *syg* genes with *mig-5* or *lin-17* (Table 2). In contrast, L1s of double mutants with either *mig-5* or *lin-17* and *fmi-1* were no different than the single-mutant backgrounds where *fmi-1* was intact. Together, we conclude that the Wnt pathway can affect neurite formation in both DDs and VDs, while the Syg pathway is DD specific and the Fmi pathway VD specific.

**Figure 5:**
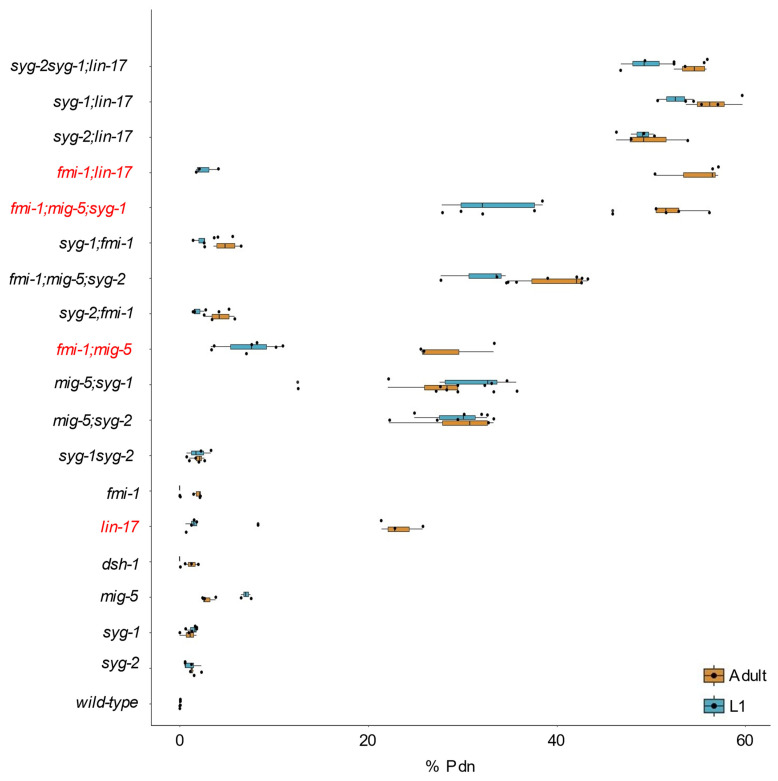
Stage-specific Pdn formation by genotype. A boxplot of the percentage of animals displaying Pdns as L1s (blue boxes) or adults (tan boxes). Genotypes observed to have a significant difference between stages are highlighted (red text).

Finally, we assessed whether the stage of scoring was significant across the genotypes. We found that only four of the genotypes, *lin-17, fmi-1;mig-5, fmi-1;mig-5;syg-1* and *fmi-1;lin-17*, exhibited statistically significant differences between Pdns observed in L1 and adults, and in all of these cases, the number of Pdns was greater in the adults compared to L1s (Figure 5, Supplemental Table S3). 

### 3.5. syg-2 and Wnt Signaling Mutations Result in Bipolar DD Neurons

During the early phase of axon initiation, both anterior and posterior processes begin to form, but only the anterior process continues to extend, leading to a single mature neurite that is anterior to the cell body [31]. However, in our analysis of L1 animals, we observed DD6 neurons with both a posterior and an anterior neurite (Figure 4D–F). We also found this could also occur in *mig-5* animals (Figure 4G–I) and in the *syg-2;mig-5* double mutants (Figure 4J–L). Thus, in these cases, the DD cell in question was converting from a pseudounipolar neuron to a bipolar morphology. This was another distinction from the effects of the *fmi-1* mutations, where VD neurons with Pdns formed only one process, but going in the posterior direction [1].

We examined the rate of Pdn formation in the *syg-2* mutants specifically for the incidence of unipolar or bipolar Pdns (Table 3). In L1s, we found that all of the animals observed with Pdns had a DD6 that formed both an anterior and posterior process. In adults, there are times when the posterior cluster is sufficiently separated to visualize each cell body individually (see Figure 3D). In those adults where it was possible to see DD6, we found that the Pdns were bipolar (note that one of the Pdns scored could not be unambiguously scored for polarity, because the neurons were clustered together). We reexamined *syg-2; mig-5* L1s, and all of the Pdns observed were bipolar. In at least one instance (Figure 4K,L), the commissure formed supernumerary branches.

We also scored the rate of unipolar vs. bipolar Pdns for the *lin-17; syg-2* double mutants (Table 3). In the L1 animals, the Pdns scored appeared to be entirely bipolar, where DD6 forms two branches. Interestingly, in the adults, we found that, while the rate of Pdn formation is consistent (~35%), some of the animals had unambiguously unipolar Pdns as adults. We then reviewed the rate of formation for the *mig-5; syg-2* doubles. Again, in L1s, all of the Pdns were observed to be bipolar, but in the doubles, there was a fraction of Pdns that were unambiguously unipolar. This would suggest that in the Wnt signaling mutants observed (*mig-5* and *lin-17*), some neurons might have resolved one of these branches, or that these are Pdns from VDs affected by the Wnt signaling pathway. We also noted that in *mig-5dsh-1* double-mutant adults, 100% of the Pdns observed were bipolar (Table 3).

### 3.6. syg-2 Mutations Result in Fewer DD Synapses

**Figure 6:**
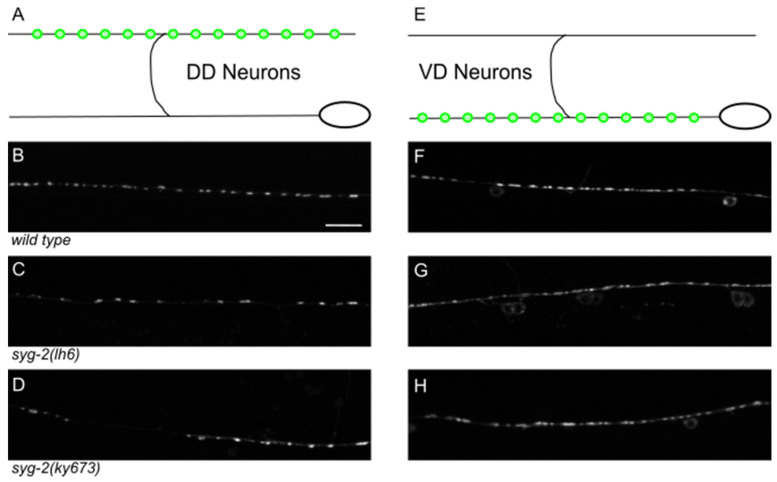
*syg-2* mutants have fewer DD synapses. (**A**) The DD neurons form presynaptic puncta (illustrated by green circles) along the dorsal nerve cord. (**B**) In the wild-type dorsal nerve cord, synaptic puncta are regularly sized and spaced along the neuron process. (**C**,**D**) In *lh6* (**C**) or *ky673* (**D**) animals, fewer synaptic puncta are formed, with gaps and irregular spacing. (**E**) VD neurons form puncta along the ventral nerve cord. (**F**) A wild-type ventral nerve cord. (**G**,**H**) In the synapses formed in *lh6* (**G**) or *ky673* (**H**), the number of synapses is roughly equivalent to the wild type.

Finally, because of a previous observation that *syg-2* mutants result in the aberrant formation of HSN synapses, we examined a presynaptic marker (*juIs1*) in the *syg-2* mutant animals. The DD neurons are presynaptic to the dorsal body wall muscles, while the VDs innervate ventral body wall muscles. Therefore, we specifically examined the pattern of SNB-1::GFP in the dorsal nerve cord of day 1 adults (Figure 6).

We observed that the size and shape of synaptic puncta were generally normal, albeit slightly enlarged (Figure 4, Table 4). There did appear to be a reduction in the total number of puncta formed, and we were able to observe regions of the axons with large gaps, at a frequency much higher than that in the wild type. The relatively mild effects on synapses in the DD neurons are at odds with the completely penetrant phenotype observed in the HSNs, but consistent with the size of the effect *syg-2* mutants have on neurite development alone.

## 4. Discussion

Here, we demonstrate that the IgSF genes *syg-1* and *syg-2* have roles in the development of neurites emerging from the DD GABAergic neurons. This adds a new function to these proteins, which have largely been studied for their function in synaptogenesis [17,18,19,32]. In previous reports, SYG-1 was identified to function cell autonomously within the HSNs as a receptor for SYG-2, which functioned on adjacent vulval epithelial cells to instruct the HSNs where synapses will be needed later in development. Interestingly, neither *syg-1* nor *syg-2* mutants are reported to have gross HSN axon outgrowth defects, suggesting the functions in those cells are synapse specific. This would also be consistent with the appearance of SYG-1, in the HSNs, just before the formation of synapses, but well after axon pathfinding is complete [18].

As had been previously reported for the HSNs, we found no gross changes in the specification or cell division of GABAergic neurons or their precursors, nor were there obvious migration errors in cell positioning. Thus, we conclude that early developmental events are either independent of these genes or are compensated by other molecules. In either case, the absence of earlier developmental defects enabled us to focus on the role of these proteins in axonal development.

The DD and VD neurons share a common morphology, despite being born at different times and coming from different cell lineages. They are also distinct from the other *C. elegans* motorneuron morphologies, some of which do have two distinct processes (see Figure 1 [31]). However, we have found that both the DDs and VDs rely on the Wnt signaling pathway to regulate normal axon outgrowth. In animals with mutations in the Wnt signaling pathway, we found defects in axon outgrowth, including Pdns, left/right defects and axon termination errors [33]. The incomplete penetrance of Pdns indicates that other pathways can compensate when Wnt signaling is perturbed. However, we found here that the DD neurons appear to use the Syg pathway, while the VD neurons specifically rely on the Fmi pathway. All of our analyses seem to concur that each genetic pathway is both cell type specific and independent.

Another difference between our observations of Pdns here and our previous report is the identification of GABAergic neurons with a bipolar morphology. In our studies of *fmi-1* mutants, we found Pdns formed by a “reversal” of the cell’s neurite extension program, resulting in a neuron with a single process that projects in the incorrect direction. This observation is largely suggestive of guidance errors leading to the observed Pdns. In contrast, here, we found that in the Syg pathway mutants with Pdns, primarily the neurons extend both an anterior and posterior process. The Wnt pathway mutants appear to result in both bipolar and unipolar Pdns, perhaps also by cell type, as in L1s, we generally see bipolar morphologies, but adults are mixed. This suggests that there is an additional, non-guidance error, in initial outgrowth, with the posteriorly directed neurite extending inappropriately.

Previous work has shown that Wnt components are important for polarization of neuronal compartments, and even some Wnt pathway proteins are themselves polarized inside neurons [34,35,36]. Wnt mutants exhibit altered accumulation of synaptic vesicles to non-synaptic domains, suggesting trafficking defects. Similarly, in the HSNs of *syg-1* or *syg-2* mutants, synaptic vesicles are transported to an incorrect position within the axon. Altogether, these data suggest that during the early stages of neurite development, incorrectly trafficked membrane-associated proteins or vesicles could be contributing to the observed aberrant growth. It is worth noting that during the earliest stages of axon initiation in GABAergic neurons, two processes begin to form normally. This is true by light microscopy for VDs [1] and is described in DDs by ultrastructural analysis of embryonic neural development (see Figure 3.4 in [37]). However, at a very early stage, the posterior process growth ceases, while the anterior growth proceeds, leading to mature neurons with only a single anterior process. From this, we conclude that, in wild-type animals, the Syg, Fmi and Wnt pathways normally function to bias the growth toward the anterior. 

We also probed different components of the Wnt signaling pathway, focusing here on one Frizzled-like receptor, LIN-17, and two Disheveled proteins, MIG-5 and DSH-1. Our results suggest that *mig-5* and *dsh-1* are compensatory, which has been shown to be true in other contexts as well. Further, we found that mutations in these two genes differentially affected Pdns in the mutant backgrounds we analyzed. Most specifically, loss of *mig-5* is synergistic with *fmi-1,* while loss of *dsh-1* is approximately additive. Taken together, these results argue that the Disheveled proteins function in discrete roles in neurite formation in D-type neurons. 

It is curious that so many different genes contribute to the formation of these neurons. Our data suggest that the loss of function in most of the genes analyzed results in a low incidence of Pdns. It is only upon mutant combinations that we begin to identify high rates of errors in extension in the inappropriate direction. The role of the D-type neurons is to provide inhibitory input to the locomotor circuit, and, presumably, errors in outgrowth would result in inefficiencies in locomotion; therefore, there are redundant pathways that have developed to compensate within these cells to ensure the fidelity of axon development. Further work will be required to understand the consequences of these axon outgrowth errors more completely in these animals.

## Figures and Tables

**Table 1:** Pdns observed in adults (DDs and VDs).

Genotype	Pdns	Total	Average	St. Dev.	Additive ^a^	*p* Value (Comparison)
*wild-type*	0	362	0.0%	0.0%	ND	
*mig-5(rh94)*	9	323	2.9%	0.7%	ND	0.011 (wt)
*syg-2(lh6)*	7	504	1.3%	0.2%	ND	0.046 (wt)
*mig-5;syg-2(lh6)*	234	814	29.5%	4.2%	4.1%	<0.001 (additive)
*syg-2(ky673)*	26	1094	2.1%	1.2%	ND	0.001 (wt)
*mig-5;syg-2(ky673)*	106	391	27.1%	1.9%	4.9%	<0.001 (additive)
*syg-1(ky652)*	8	735	1.0%	0.8%	ND	
*mig-5;syg-1*	129	451	28.7%	1.3%	3.9%	<0.001 (additive)
*syg-2(lh6);syg-1*	8	406	2%	1%	2.3%	1.00 (additive)
*lin-17(n671)*	63	273	23.3%	2.2%	ND	<0.001 (wt)
*syg-2(lh6);lin-17*	131	260	49.8%	3.9%	24.3%	<0.001 (additive)
*syg-1;lin-17*	297	528	56.5%	2.6%	24.1%	<0.001 (additive)
*syg-2(lh6)syg-1;lin17*	302	559	54.4%	1.7%	24.8%	<0.001 (additive)
*fmi-1(tm306)*	9	459	1.9%	0.4%	ND	0.006 (wt)
*mig-5;fmi-1*	153	527	28.3%	4.4%	4.8%	<0.001 (additive)
*fmi-1;syg-2(lh6)*	33	780	4.3%	1.3%	3.2%	0.349 (additive)
*fmi-1;syg-1*	31	607	4.9%	1.3%	2.9%	0.079 (additive)
*fmi-1;mig-5;syg-2(lh6)*	343	866	40.0%	3.5%	29.2%	<0.001 (additive)
*fmi-1;mig-5;syg-1*	292	570	51.4%	3.7%	29.0%	<0.001 (additive)
*dsh-1(ok1445)*	8	560	1.3%	0.7%	ND	0.026 (wt)
*dsh-1mig-5*	234	671	34.9%	0.0%	4.2%	<0.001 (additive)
*dsh-1;lin-17*	74	321	24.2%	6.0%	24.3%	0.781 (additive)
*dsh-1;fmi-1*	30	501	6.2%	1.7%	3.2%	0.049 (additive)
*lin-17;fmi-1*	256	479	54.7%	3.7%	24.8%	<0.001 (additive)
*dsh-1;lin-17;fmi-1*	258	450	57.9%	2.0%	28.9%	<0.001 (additive)

^a^ Predicted additive phenotype (P1 + P2 − (P1 × P2)).

**Table 2:** Pdns observed in L1s (DDs).

Genotype	Pdns	Total	Average	St. Dev.	Additive ^a^	*p* Value (Comparison)
*wild-type*	0	416	0.0%	0.0%	ND	
*mig-5(rh94)*	15	219	7.0%	0.7%	ND	<0.0001 (wt)
*syg-2(lh6)*	11	1055	1.1%	1.1%	ND	0.041 (wt)
*mig-5;syg-2(lh6)*	148	516	29.2%	3.9%	8.0%	<0.0001 (additive)
*syg-2(ky673)*	19	1294	1.4%	0.2%	ND	0.007 (wt)
*mig-5;syg-2(ky673)*	140	409	34.2%	2.0%	8.3%	<0.0001 (additive)
*syg-1(ky652)*	17	1136	1.3%	0.7%	ND	
*mig-5;syg-1*	228	702	32.1%	3.1%	8.2%	<0.0001 (additive)
*syg-2(lh6);syg-1*	8	464	1.9%	1.3%	2.4%	0.644 (additive)
*fmi-1(tm306)*	0	585	0.0%	0.0%	ND	1.000 (wt)
*mig-5;fmi-1*	51	754	7.3%	2.9%	7.0%	0.919 (additive)
*fmi-1;syg-2(lh6)*	13	729	1.9%	0.6%	1.1%	0.380 (additive)
*fmi-1;syg-1*	12	563	2.2%	0.7%	1.3%	
*fmi-1;mig-5;syg-2(lh6)*	189	597	31.9%	3.7%	8.3%	<0.0001 (additive)
*fmi-1;mig-5;syg-1(ky652)*	264	804	33.2%	4.7%	8.5%	<0.0001 (additive)
*lin-17* *(n671)*	54	1111	2.7%	3.2%	ND	<0.0001 (wt)
*dsh-1(ok1445)*	0	188	0%	0.0%	ND	1.000 (wt)
*dsh-1(ok1445)mig-5(tm2639)*	42	157	27%	0.0%	7.0%	<0.0001 (additive)
*syg-2(lh6);lin-17*	103	207	49.1%	1.7%	2.4%	<0.0001 (additive)
*syg-1;lin-17*	172	325	52.6%	2.7%	2.6%	<0.0001 (additive)
*syg-2(lh6)syg-1;lin17*	383	775	49.5%	2.8%	3.2%	<0.0001 (additive)
*lin-17;fmi-1*	13	401	2.6%	1.3%	2.7%	0.997 (additive)

^a^ Predicted additive phenotype (P1 + P2 − (P1 × P2)).

**Table 3:** Pdn type.

Genotype	Pdns	Total	Unipolar	Bipolar	Ambiguous	Bipolar (%)
L1						
*syg-2(lh6)*	7	454	0	7	0	100%
*syg-2(lh6);mig-5(rh94)*	110	766	0	110	0	100%
*lin-17(n671)*	47	567	0	47	0	100%
*lin-17(n671);syg-2(lh6)*	318	913	4	314	0	99%
*dsh-1(ok1445)mig-5(tm2639)*	42	157	0	42	0	100%
Adult						
*syg-2(lh6)*	3	373	0	2	1	67%
*syg-2(lh6);mig-5(rh94)*	139	558	31	69	39	50%
*lin-17(n671)*	60	417	31	29	0	48%
*lin-17(n671);syg-2(lh6)*	104	295	13	40	51	38%
*dsh-1(ok1445)mig-5(tm2639)*	234	671	0	234	0	100%

**Table 4:** Synaptic measurements.

Genotype	Area (Avg ± SD) μm^2^	N	*p* (vs. Wild Type)	Puncta (Avg ± SD) #/100 μm	N	*p* (vs. Wild Type)
Ventral Cord (VD Neurons)						
*wild type*	1.12 ± 1.17	517	-	24.0 ± 2.9	11	-
*syg-2(lh6)*	1.53 ± 2.11	402	4 × 10^−4^	21.9 ± 2.3	9	0.09
Dorsal Cord (DD Neurons)						
*wild type*	0.68 ± 0.41	326		21.9 ± 4.7	9	
*syg-2(lh6)*	1.01 ± 1.16	214	1 × 10^−4^	14.1 ± 3.9	8	0.002

## Supplementary Materials

The following supporting information can be downloaded at: https://www.mdpi.com/article/10.3390/jdb10010003/s1, Table S1: Significance testing of *syg-2* by genotype; Table S2: Significance testing of *syg-1, syg-2, mig-5* and *lin-17*; Table S3: Significance testing of L1 vs. adult.
