# Supplementary material for: The Immunoglobulin Superfamily Members syg-2 and syg-1 Regulate Neurite Development in C. elegans
Source: J Dev Biol. 2022 Jan 9;10(1):3. doi: 10.3390/jdb10010003 (PMC8788504; doi:10.3390/jdb10010003)
Supplement: Supplementary file 1 [file jdb-10-00003-s001.zip › jdb-1306365-supplementary.pdf]

Supplemental Tables

Supplemental Table S1. Significance testing of *syg-2* by genotype.

**ANOVA**						
	**Df**	**Sum of Sq**	**Mean of Sq**	**F Value**	**Pr (>F)**	**Significant?**
Genotype	3	4123	1374.5	235.969	1.23×10^{-14}	Yes
Allele	1	3	2.7	0.455	0.508	No
Genotype&Allele	1	11	10.8	1.856	0.19	No
Residuals	18	105	5.8			
Tukey HSD		Padj	Significant?			
Genotype 1	Genotype 2					
wild type	*syg-2(lh6)*	1.00	No			
	syg-2(ky673)	0.99	No			
	mig-5	0.97	No			
	mig-5;syg-2(lh6)	1.04×10^{-10}	Yes			
	mig-5;syg-2(ky673)	5.21×10^{-9}	Yes			
syg-2(lh6)	*syg-2(ky673)*	1.00	No			
	mig-5;syg-2(lh6)	2.29×10^{-10}	Yes			
	mig-5;syg-2(ky673)	1.13×10^{-8}	Yes			
syg-2(ky673)	*mig-5;syg-2(ky673)*	1.87×10^{-9}	Yes			
mig-5	*syg-2(lh6)*	1.00	No			
	syg-2(ky673)	1.00	No			
	mig-5;syg-2(lh6)	6.48×10^{-10}	Yes			
	mig-5;syg-2(ky673)	3.26×10^{-8}	Yes			
mig-5;syg-2(lh6)	*syg-2(ky673)*	1.05×10^{-11}	Yes			
	mig-5;syg-2(ky673)	0.98	No			

Supplemental Table S2. Significance testing of *syg-1, syg-2, mig-5* and *lin-17*.

**ANOVA**						
	**Df**	**Sum of Sq**	**Mean of Sq**	**F Value**	**Pr (>F)**	**Significant?**
Genotype	8	4.12×10^{8}	1912.7	291.3	$<2 \times 10^{-16}$	Yes
Residuals	25	164	6.6			
Tukey HSD						
Genotype 1	Genotype 2	Padj	Significant?			
syg-2	*syg-1*	1.00	No			
	syg-1syg-2	1.00	No			
	mig-5;syg-2	7.64×10^{-13}	Yes			
	mig-5;syg-1	3.49×10^{-11}	Yes			
	lin-17	3.67×10^{-9}	Yes			
	syg-1;lin-17	3.28×10^{-14}	Yes			
syg-1	*mig-5;syg-2*	1.11×10^{-13}	Yes			
	mig-5;syg-1	6.39×10^{-12}	Yes			
	lin-17	7.16×10^{-10}	Yes			
syg-1syg-2	*syg-1*	1.00	No			
	mig-5;syg-2	2.01×10^{-13}	Yes			
	mig-5;syg-1	1.40×10^{-11}	Yes			
	lin-17	1.78×10^{-9}	Yes			
	syg-1;lin-17	3.28×10^{-14}	Yes			
mig-5;syg-2	*mig-5;syg-1*	1.00	No			
	lin-17	4.58×10^{-2}	Yes			
mig-5;syg-1	*lin-17*	0.24	No			

syg-2;lin-17	*syg-2*	3.28 × 10^{-14}	Yes
	syg-1	3.28 × 10^{-14}	Yes
	syg-1syg-2	3.28 × 10^{-14}	Yes
	mig-5;syg-2	1.06 × 10^{-9}	Yes
	mig-5;syg-1	9.21 × 10^{-9}	Yes
	lin-17	7.70 × 10^{-11}	Yes
	syg-1;lin-17	4.70 × 10^{-2}	Yes
syg-1;lin-17	*syg-1*	3.28 × 10^{-14}	Yes
	mig-5;syg-2	3.09 × 10^{-13}	Yes
	mig-5;syg-1	6.21 × 10^{-12}	Yes
	lin-17	1.49 × 10^{-13}	Yes
syg-2syg-1;lin17	*syg-2*	3.28 × 10^{-14}	Yes
	syg-1	3.28 × 10^{-14}	Yes
	syg-1syg-2	3.28 × 10^{-14}	Yes
	mig-5;syg-2	1.73 × 10^{-12}	Yes
	mig-5;syg-1	3.61 × 10^{-11}	Yes
	lin-17	5.33 × 10^{-13}	Yes
	syg-2;lin-17	0.36	No
	syg-1;lin-17	0.96	No

Supplemental Table S3. Significance testing of L1 vs. adult.

**ANOVA**						
	**Df**	**Sum of Sq**	**Mean of Sq**	**F value**	**Pr (>F)**	**Significant?**
Genotype	21	53843	2564	51.23	2.00 × 10^{-16}	Yes
Stage	1	1752	1752.1	35.01	2.00 × 10^{-16}	Yes
Genotype&Stage	18	5139	285.5	26.41	2.00 × 10^{-16}	Yes
Residuals	108	1168	10.8			

Tukey HSD				
Genotype	Stage		p (adjusted)	Significant?
wild-type	L1	Adult	1.00	No
syg-2	L1	Adult	1.00	No
syg-1	L1	Adult	1.00	No
syg-1syg-2	L1	Adult	1.00	No
mig-5	L1	Adult	1.00	No
mig-5;syg-2	L1	Adult	1.00	No
mig-5;syg-1	L1	Adult	1.00	No
lin-17	L1	Adult	6.52 × 10^{-11}	Yes
syg-2;lin-17	L1	Adult	1.00	No
syg-1;lin-17	L1	Adult	1.00	No
syg-2syg-1;lin-17	L1	Adult	1.00	No
fmi-1	L1	Adult	1.00	No
mig-5;fmi-1	L1	Adult	2.63 × 10^{-12}	Yes
fmi-1;syg-2	L1	Adult	1.00	No
fmi-1;mig-5;syg-2	L1	Adult	0.20	No
fmi-1;syg-1	L1	Adult	1.00	No
fmi-1;mig-5;syg-1	L1	Adult	2.60 × 10^{-11}	Yes
lin-17;fmi-1	L1	Adult	1.15 × 10^{-14}	Yes
dsh-1	L1	Adult	1.00	No
